# Identification of a microRNA signature associated with survivability in cervical squamous cell carcinoma

**DOI:** 10.1371/journal.pone.0193625

**Published:** 2018-03-07

**Authors:** Chengbin Ma, Wenying Zhang, Qiongwei Wu, Yu Liu, Chao Wang, Guoying Lao, Longtao Yang, Ping Liu

**Affiliations:** Department of Gynecology, Changning Maternity and Infant Health Hospital, Shanghai, China; Duke Cancer Institute, UNITED STATES

## Abstract

**Background:**

The aim of this study is to find the potential miRNA expression signature capable of predicting survival time for cervical squamous cell carcinoma (CSCC) patients.

**Methods:**

The expression of 332 miRNAs was measured in 131 (Training cohort) and 130 (Validation cohort) patients with CSCC in the Cancer Genome Atlas (TCGA) data portal. The miRNA expression signature was identified by Cox Proportion Hazard regression model to the Training data set, and subsequently validated in an independent Validation set. Kaplan-Meier curves and the receiver operating characteristic analyses of 5 years were used to access the overall survival of miRNA signature. MiRNA signature-gene target analysis was performed, followed by the construction of the regulatory network. Gene Ontology and Kyoto Encyclopedia of Genes and Genomes pathway analysis were used to explore the function of target genes of miRNA signature.

**Results:**

A 2-miRNA expression signature of hsa-mir-642a and hsa-mir-378c associated with survivability was identified in CSCC. Both of them had a significant diagnostic and prognostic value of patients with CSCC. A total of 345 miRNA signature-target pairs were obtained in the miRNA signature-gene target regulatory network, in which 316 genes were targets of has-mir-378c and has-mir-642a. Functional analysis of target genes showed that MAPK signaling pathway, VEGF signaling pathway and endocytosis were the significantly enriched signal pathways that covered most genes.

**Conclusions:**

The 2-miRNA signature adds to the prognostic value of CSCC. In-depth interrogation of the 2-miRNAs will provide important biological insights that finding and developing novel molecularly prediction to improve prognosis for CSCC patients.

## Introduction

Cervical squamous cell carcinoma (CSCC), accounting for about 75–80% of all cervical cancers, is one of the most common gynecological malignancy and leads to the cancer death in women [1, 2]. Walboomers JM and Castellsague X et al found that CSCC was closely associated with high-risk human papillomavirus (HPV) infection [3, 4]. In addition, lymph node metastasis is one of diffusion routes that influence survival and prognosis of CSCC [5, 6]. Once lymph node metastasis occurs, the overall 5-year survival rate for early stage carcinoma of the uterine cervix is reduced to 53%, which lead to the high recurrence rate and poor prognosis of patients with CSCC [7–10]. As there are no valid diagnostic and therapeutic methods for CSCC, it is urgent to understand the pathological mechanism and find potential biological markers for diagnosis, therapy and prognosis of patients with CSCC. [11, 12]. Several genes have been identified as the diagnostic and prognostic biomarkers for CSCC. It has been demonstrated that the kinase family member 20a (KIF20A) protein is one potential biomarker for CSCC [13]. Additionally, Liu DQ et al suggested that receptor interacting serine/threonine kinase 4 (RIPK4) might act as a potential diagnostic and independent prognostic biomarker for patients with CSCC [14].

MicroRNAs (miRNAs) are small non-coding RNAs that are approximately 22 nt in size. They can modulate growth, proliferation, differentiation and apoptosis of cells by regulating target genes expression at the post-transcriptional level. As microRNAs stably present in almost all body fluids, they constitute a new class of non-invasive biomarkers [15–19]. It has been reported that the deregulation of miRNAs leads to the occurrence of a number of diseases, such as cancers in cervical [20]. MiR-23b/uPA is involved in the HPV-16 E6-associated cervical cancer development [21]. MiR-372 is down-regulated in cervical cancer tissues compared with normal cervical tissues [22]. The down-regulation of miR-143 is associated with lymph node metastasis and poor prognosis in cervical cancer [23, 24]. It is reported that 6 serum microRNAs including miR-1246, miR-20a, miR-2392, miR-3147, miR-3162-5p and miR-4484 has been identified in predicting lymph node metastasis of CSCC patients [25]. In addition, it is found that serum miR-206 is a powerful tool to predict chemoradiotherapy sensitivity in advanced-stage CSCC patients [26]. It is noteworthy that the identification of potential miRNAs that participate in survival prediction is essential for establishing novel prognosis strategies for CSCC. Recently, miRNA expression signatures related to prognosis have been found in number of malignancy [27]. Hence, we undertook to identify and validate a miRNA expression signature capable of predicting for survivability in CSCC patients.

## Material and methods

### TCGA data retrieval and analysis

The BCGSC__IlluminaHiSeq_miRNASeq data were acquired from Firebrowse (http://firebrowse.org/?cohort=LIHC&download_dialog=true, 2016-01-28). Level 3 (Reads-per-kilobase-million; RPKM) miRNA-Seq and Level 1 clinical data were downloaded from the TCGA data portal (http://tcga-data.nci.nih.gov/tcga) dataset. At the time of analysis, there were 307 clinical histories. Only those clinical histories with miRNA-seq values and sufficient follow-up data (261 cases) were used for further survival analysis. All these cases were randomly divided into Training cohort (131 cases) and Validation cohort (130 cases). There was no significant difference in gender, race, family history, tumor stage, vascular invasion, follow-up time and follow-up result between two cohorts. Clinical characteristics for two cohorts were shown in Table 1.

**Table 1:** The clinical characteristics of the patients in the two cohorts.

Factor		All cohorts (n = 261)	Training cohorts (n = 131)	Validation cohorts (n = 130)	p-value
**Age**	Mean±SD	48.77±13.49	48.52±13.12	49.01±13.91	0.7741
	Median	47	47	47	
**Race**	Asian	15	10	5	0.3705
	White	185	88	97	
	Black or African American	24	13	11	
	Native hawaiian or other pacific islander	2	2	0	
	American indian or Alaska native	8	4	4	
**Tumor grade**	G1	15	7	8	0.7578
	G2	115	54	61	
	G3	106	56	50	
	G4	1	0	1	
	Gx	21	10	11	
**Stage**	I	136	68	68	0.4456
	II	61	34	27	
	III	40	21	19	
	IV	20	7	13	
**Lymphovascular** **Invasion**	present	71	35	36	0.8577
	absent	65	34	31	
**Vital status**	Alive	201	101	100	0.4828
	Dead	60	30	30	
**Survival time**	Mean±SD	1032.85±1137.81	1054±1093.37	1011.53±1184.76	0.7637
	Median	607	659	601.5	

### Identification and survival analysis of miRNA signature

In order to identify the survival time related miRNAs in CSCC, the single factor Cox proportional hazard (CoxPH) regression model was fitted to the Training cohort data. The statistical significance was set at p<0.05. After further adjustment, the multi-factor CoxPH regression model was used for identification of miRNA signature in the survival evaluation model of CSCC. A risk score (RS) was calculated using the coefficients from the model, and high *vs*. low risk patients were then compared in the Training cohort and Validation cohort using the log-rank test. Kaplan-Meier curves were used to plot overall survival with miRNA signature expression using Cutoff Finder (http://molpath.charite.de/cutoff). In addition, the receiver operating characteristic (ROC) analyses were performed to assess the 5 years’ survival rate of miRNA signature of CSCC by using pROC package in R language. The area under the curve (AUC) under binomial exact confidence interval was calculated and the ROC curve was generated.

### Network construction of miRNA signature-targets

Identifying target genes is an important step in studying the function of miRNA in tissues. In this study, target genes of miRNA signature were obtained by miRWalk (http://www.umm.uni-heidelberg.de/apps/zmf/mirwalk/). According to the miRNA-target pairs, miRNA signature-targets interaction network was established by Cytoscape software (http://www.cytoscape.org/).)

### Functional annotation of miRNA signature targets

In order to study the biological function of target genes of miRNA signature, the Gene Ontology (GO) and Kyoto Encyclopedia of Genes and Genomes (KEGG) pathway analysis were performed by using the online software GeneCodis3 (http://genecodis.cnb.csic.es/analysis). The threshold of false discovery rate (FDR) < 0.05 was set as the criteria of statistical significance.

## Results

### Generation and validation of miRNA signature

The single factor CoxPH regression model fitted to the Training cohort yielded 47 miRNAs (Table 2). A group of miRNA signatures including hsa-mir-642a and hsa-mir-378c (Table 3) was identified that were most strongly associated with survival after multi-factor CoxPH regression model analysis. The miRNA signature was combined with their coefficients within the penalized model to yield the following equation:

**Table 2:** The single factor CoxPH regression model fitted to the Training cohort.

miRNA	Coefficient	HR	95%CI lower	95%CI upper	p-value
hsa-mir-361	-0.741962146	0.476178666	0.337831316	0.671181479	2.27E-05
hsa-mir-150	-0.399833202	0.670431863	0.544302388	0.825788924	1.70E-04
hsa-mir-642a	-0.933407714	0.393211473	0.241114727	0.64125184	1.84E-04
hsa-mir-142	-0.459467464	0.631619916	0.493836414	0.807845893	2.53E-04
hsa-mir-378c	-0.704272826	0.494468008	0.330219908	0.74041148	6.28E-04
hsa-mir-148b	-0.940559471	0.390409352	0.218272292	0.698299636	1.52E-03
hsa-mir-502	-0.823303663	0.438979018	0.259043417	0.743900693	2.22E-03
hsa-mir-532	-0.689571509	0.501791036	0.32109448	0.784174938	2.47E-03
hsa-mir-548o	-1.431560505	0.238935771	0.093023518	0.613719019	2.94E-03
hsa-mir-629	-0.582879033	0.558288719	0.378627906	0.823199476	3.26E-03
hsa-mir-3607	-0.455590425	0.634073485	0.465711706	0.863300575	3.81E-03
hsa-mir-140	-0.831986343	0.435184003	0.245859481	0.770298201	4.29E-03
hsa-mir-653	-0.559648681	0.571409776	0.384603247	0.848950534	5.59E-03
hsa-mir-3940	-0.798489446	0.450008213	0.255437141	0.792787577	5.72E-03
hsa-mir-204	-0.503949703	0.604139769	0.417830953	0.87352279	7.39E-03
hsa-mir-500b	-0.689845567	0.501653535	0.302748834	0.831237782	7.42E-03
hsa-mir-659	-0.984396955	0.373664495	0.177043262	0.788649926	9.80E-03
hsa-mir-33b	-0.49164916	0.611616908	0.421114439	0.888298305	9.82E-03
hsa-mir-331	-0.661490245	0.516081673	0.311061687	0.856229823	1.04E-02
hsa-mir-550a-2	-0.713332169	0.490008682	0.282973877	0.848518284	1.09E-02
hsa-mir-3074	-0.533161438	0.58674707	0.388980125	0.885063534	1.10E-02
hsa-mir-155	-0.319675972	0.726384368	0.566546989	0.931315954	1.17E-02
hsa-mir-34a	-0.601640189	0.547912219	0.34001366	0.882928646	1.35E-02
hsa-mir-942	-0.501600699	0.605560563	0.406007929	0.903193187	1.39E-02
hsa-mir-101-1	-0.572617152	0.564047308	0.356914365	0.891388514	1.42E-02
hsa-mir-1306	-0.582090351	0.558729205	0.349290949	0.893748679	1.52E-02
hsa-mir-146a	-0.298734498	0.741756321	0.579025164	0.950221983	1.81E-02
hsa-mir-3130-1	-0.335178726	0.715210248	0.538178207	0.950476427	2.09E-02
hsa-mir-766	-0.453584546	0.635346636	0.432031166	0.93434312	2.12E-02
hsa-mir-651	-0.444810245	0.640945887	0.435193351	0.94397497	2.43E-02
hsa-mir-589	-0.602353886	0.547521315	0.323793065	0.925836971	2.46E-02
hsa-mir-580	-0.907437169	0.403557148	0.18125611	0.898498661	2.63E-02
hsa-mir-128-2	-0.488785221	0.613371052	0.397361642	0.946805146	2.73E-02
hsa-mir-153-2	-0.33037904	0.718651284	0.535409284	0.964607232	2.78E-02
hsa-mir-550a-1	-0.577582055	0.561253808	0.334287794	0.94231929	2.89E-02
hsa-mir-186	-0.704267306	0.494470737	0.262595738	0.931093978	2.92E-02
hsa-mir-16-2	-0.435460059	0.646966956	0.43718272	0.957417167	2.94E-02
hsa-mir-423	-0.630852633	0.532137889	0.29790113	0.950552733	3.31E-02
hsa-mir-188	-0.536683133	0.58468436	0.353621916	0.966726849	3.65E-02
hsa-mir-3199-2	0.733244039	2.081823182	1.043660499	4.152679692	3.74E-02
hsa-mir-128-1	-0.506262412	0.602744184	0.373877643	0.971709751	3.77E-02
hsa-mir-34c	-0.247813788	0.780505269	0.617220838	0.986986242	3.85E-02
hsa-mir-335	0.328925144	1.389473842	1.017173297	1.898041918	3.87E-02
hsa-mir-660	-0.439107775	0.644611303	0.421343551	0.986187474	4.30E-02
hsa-mir-145	-0.347777277	0.706256158	0.502311525	0.993004812	4.55E-02
hsa-mir-1468	-0.518087872	0.595658435	0.358192455	0.990554007	4.59E-02
hsa-mir-99a	-0.209225673	0.811212146	0.659987923	0.997086648	4.68E-02

HR: hazard ratio; CI: confidence interval

**Table 3:** miRNA signature in CSCC.

miRNA	Coefficient	95%CI lower	95%CI Upper	*p*-value
hsa-mir-642a	-1.318	0.142266	0.5038	4.40E-05
hsa-mir-378c	-1.006	0.205868	0.6491	5.93E-04

Risk Score = -1.318×log_2_ (RPKM of hsa-mir-642a)−1.006×log_2_ (RPKM of hsa-mir-378c).

**Fig 1:**
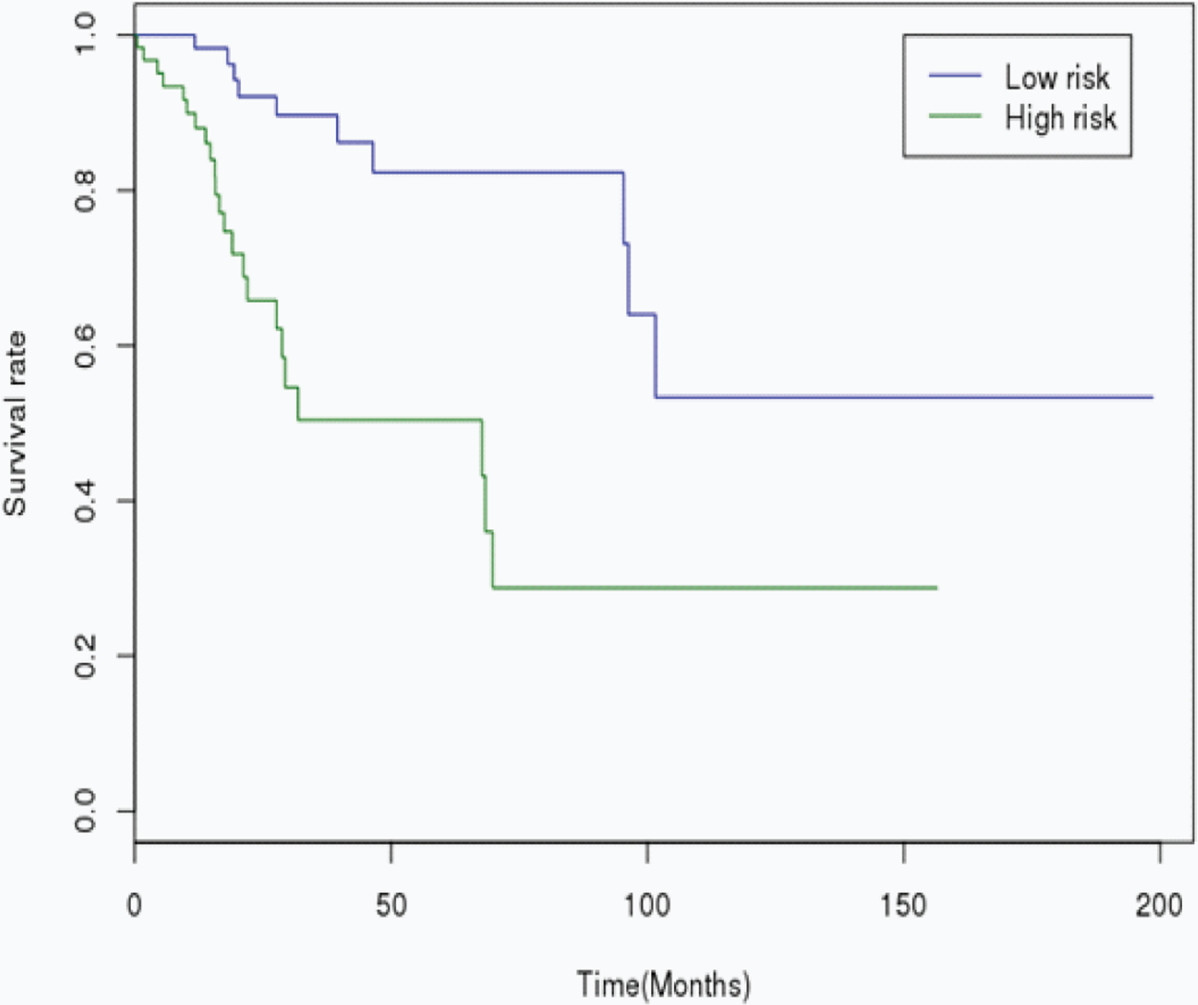
Kaplan-Meier curves showing CSCC patients dichotomized based on risk score in the Training cohort. High risk is defined as a RS ≥ the median in the training cohort, and low risk is defined as a RS < the median in the Training cohort.

**Fig 2:**
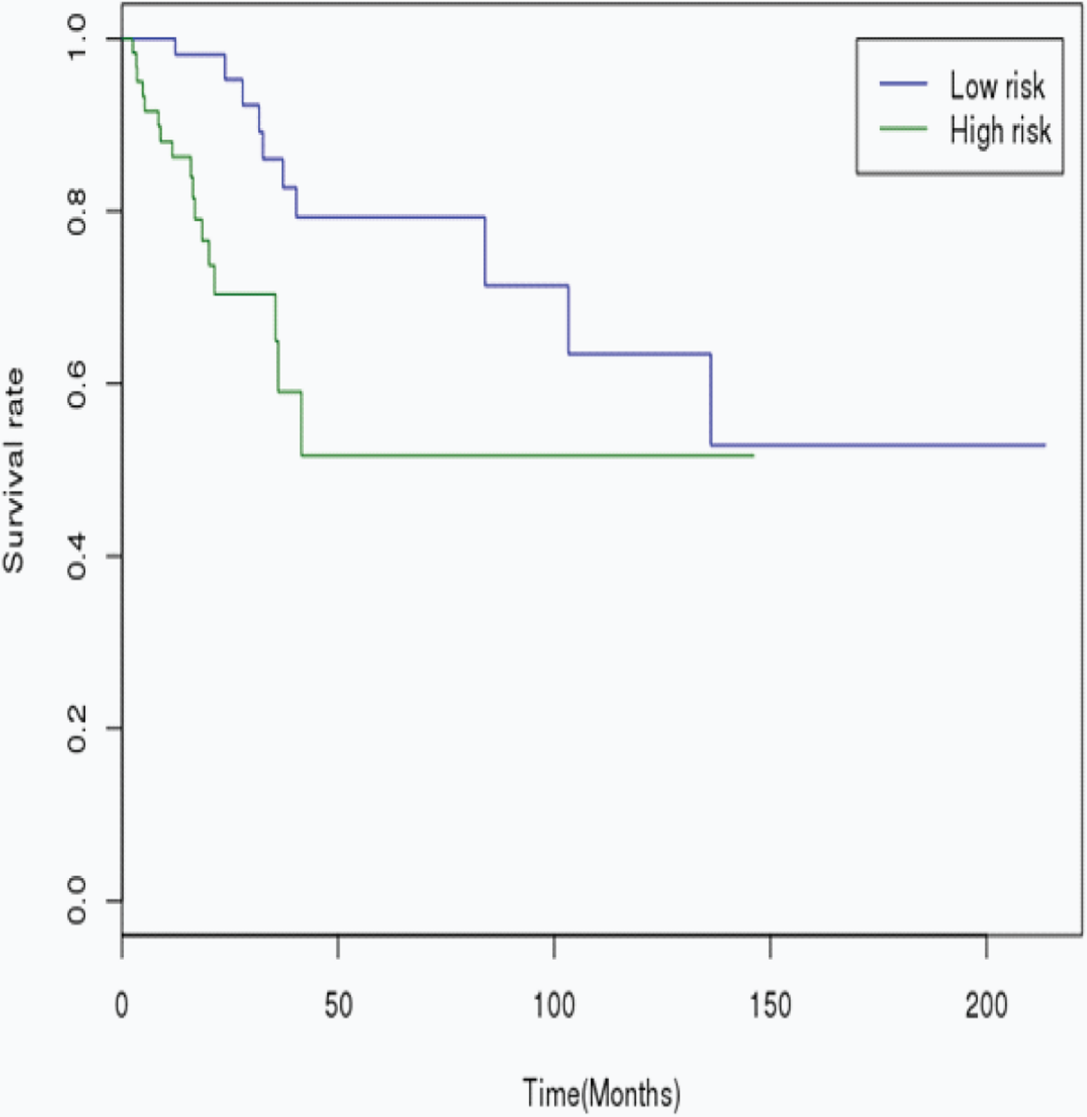
Kaplan-Meier curves showing CSCC patients dichotomized based on risk score in the Validation cohort. High risk is defined as a RS ≥ the median in the training cohort, and low risk is defined as a RS < the median in the Validation cohort.

**Fig 3:**
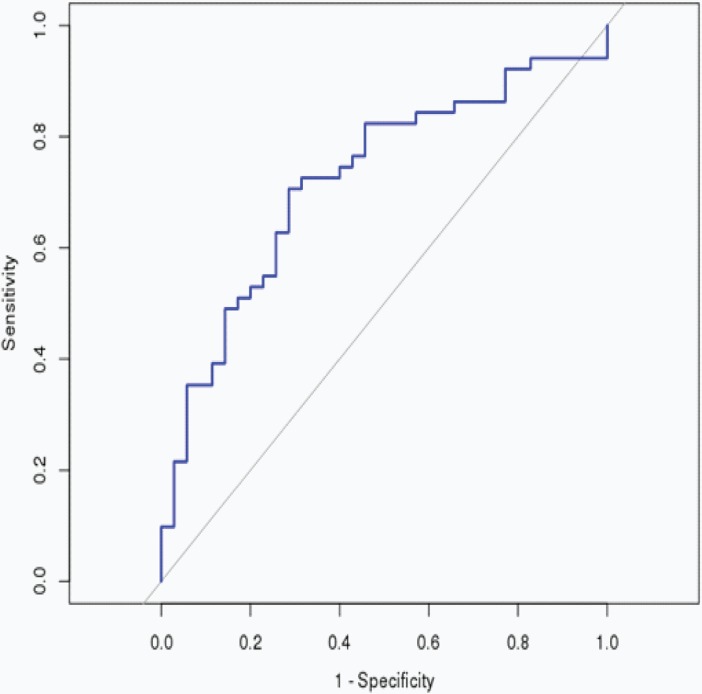
5 years’ ROC curves of miRNA signature in CSCC. The ROC curves were used to show the diagnostic ability of miRNA signature and miRNA signature with 1-Specificity (the proportion of false positive) and sensitivity (the proportion of true positive) and. The x-axis shows 1-specificity and y-axis shows sensitivity.

The RS was calculated for each patient in the Training cohort, in which the patients were dichotomized into either the “low risk” (< median), or the “high risk” (≥ median) group. A highly significant difference was observed between the high risk and the low risk group (p < 0.001), that was shown in Fig 1. When the same miRNA signature equation was applied to the Validation cohort, a similar significant difference was also observed between the high risk and the low risk group (p = 0.007), that was shown in Fig 2. Additionally, we performed 5 years’ survival analysis of miRNA signature by ROC and calculated the AUC to assess the discriminatory ability of miRNA signature (Fig 3). The AUC of the miRNA signature was 0.7221. Our result suggested that the miRNA signature could be the prognosis model for predicting the survival situation of CSCC.

### MiRNA signature-targets network

**Fig 4:**
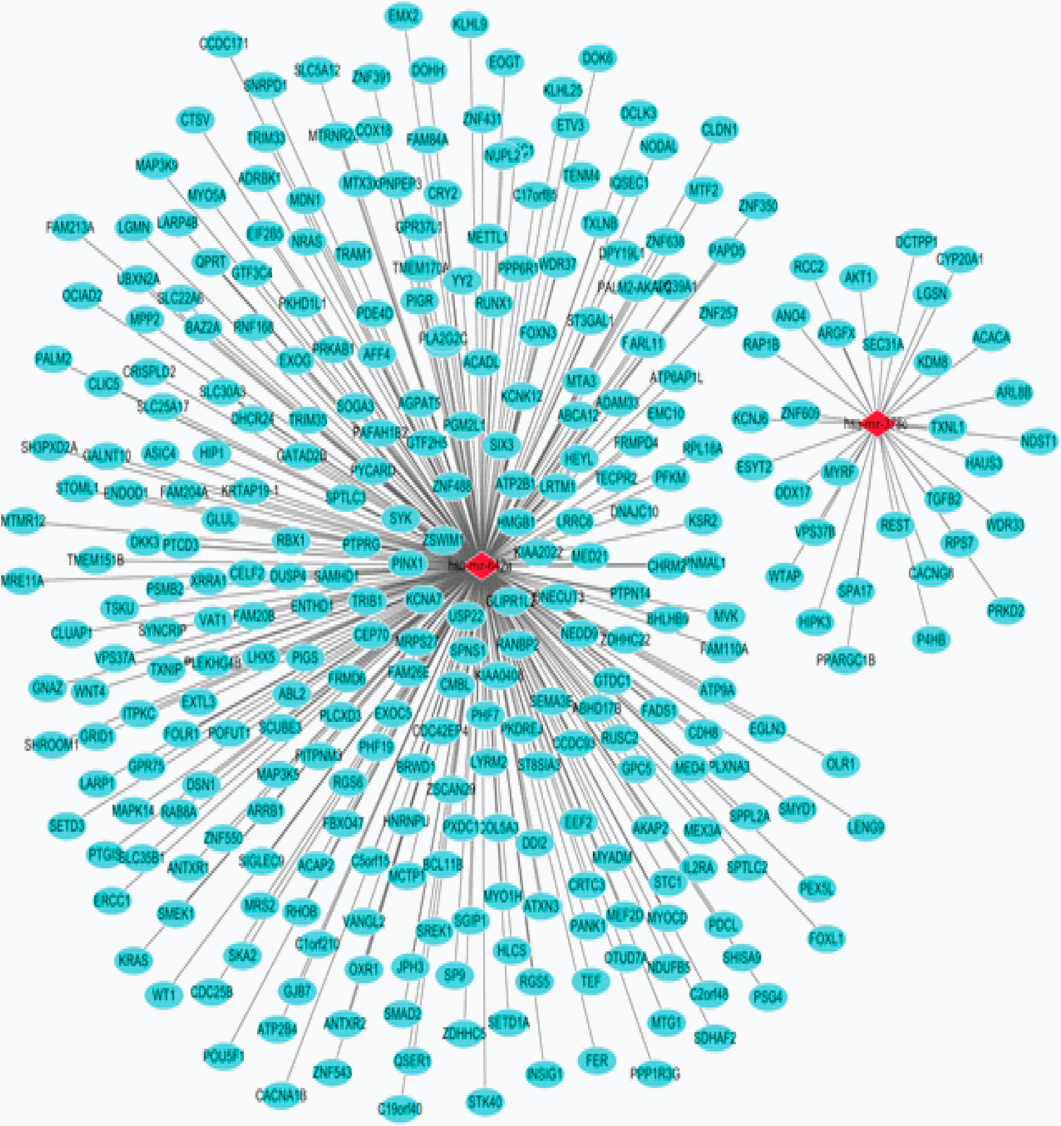
MiRNA signature-targets interaction network. The red rhombus and blue-green ellipse represented the miRNA and target genes, respectively.

A total of 345 miRNA signature-target pairs were obtained by miRWalk, followed by the construction of the interaction network (Fig 4). In the network, 316 genes were targets of has-mir-642a and has-mir-378c. The red rhombus and blue-green ellipse represented the miRNA and target genes, respectively.

### Functional annotation of miRNA signature targets

According to the GO enrichment analysis, intracellular signal transduction (FDR = 0.0001536), cell proliferation (FDR = 0.0001913) and blood coagulation (FDR = 0.0001913) were the most significantly enriched biological process; protein binding (FDR = 7.47E-12), nucleotide binding (FDR = 5.97E-09) and metal ion binding (FDR = 5.34E-08) were the most significantly enriched molecular function; nucleus (FDR = 5.68E-17), cytoplasm (FDR = 3.66E-15) and membrane (FDR = 7.10E-09) were the most significantly enriched cellular component. The top 15 GO terms were shown in Table 4. MAPK signaling pathway (FDR = 4.14E-05), VEGF signaling pathway (FDR = 8.89E-05) and endocytosis (FDR = 9.18E-05) were significantly enriched signal pathways that covered most genes. The top 15 KEGG terms were shown in Table 5.

**Table 4:** The enriched top 15 GO terms of miRNA signature targets.

GO Items	Items Details	No. of genes	FDR
**Biological process**			
GO:0035556	intracellular signal transduction	4	0.0001536
GO:0008283	cell proliferation	3	0.0001913
GO:0007596	blood coagulation	3	0.0001913
GO:0030168	platelet activation	3	0.0001913
GO:0043280	positive regulation of cysteine-type endopeptidase activity involved in apoptotic process	3	0.0001913
GO:0045087	innate immune response	3	0.0001913
GO:0007165	signal transduction	3	0.0002376
GO:0051146	nerve growth factor receptor signaling pathway	3	0.0002376
GO:0048011	striated muscle cell differentiation	3	0.0002376
GO:0045944	positive regulation of transcription from RNA polymerase II promoter	18	0.0002497
GO:0006357	regulation of transcription from RNA polymerase II promoter	6	0.0003527
GO:0018105	peptidyl-serine phosphorylation	3	0.000354
GO:0043066	negative regulation of apoptotic process	3	0.000354
GO:0007281	germ cell development	3	0.000354
GO:0006464	protein modification process	3	0.0007776
**Molecular function**			
GO:0005515	protein binding	89	7.47E-12
GO:0000166	nucleotide binding	51	5.97E-09
GO:0046872	metal ion binding	59	5.34E-08
GO:0005524	ATP binding	33	5.95E-05
GO:0046872	metal ion binding	37	6.92E-05
GO:0008270	zinc ion binding	38	0.0001139
GO:0003700	sequence-specific DNA binding transcription factor activity	7	0.0001349
GO:0003677	DNA binding	7	0.0001768
GO:0031625	ubiquitin protein ligase binding	4	0.0002863
GO:0008134	transcription factor binding	11	0.0003429
GO:0005525	GTP binding	4	0.0004299
GO:0019003	GDP binding	4	0.0004299
GO:0003924	GTPase activity	4	0.0004299
GO:0003690	double-stranded DNA binding	6	0.0005831
GO:0003697	single-stranded DNA binding	3	0.0009546
**Cellular component**			
GO:0005634	nucleus	112	5.68E-17
GO:0005737	cytoplasm	106	3.66E-15
GO:0016020	membrane	76	7.10E-09
GO:0005829	cytosol	39	1.04E-07
GO:0005730	nucleolus	38	1.10E-07
GO:0043231	intracellular membrane-bounded organelle	11	1.23E-07
GO:0016021	integral to membrane	53	8.89E-06
GO:0043231	intracellular membrane-bounded organelle	13	1.73E-05
GO:0016021	integral to membrane	68	4.08E-05
GO:0005622	intracellular	26	0.0001472
GO:0005654	nucleoplasm	14	0.0001523
GO:0005625	soluble fraction	4	0.0001675
GO:0005886	plasma membrane	4	0.0001675
GO:0005624	membrane fraction	16	0.0002292
GO:0005625	soluble fraction	8	0.000391

No.: number; FDR: false discovery rate.

**Table 5:** The enriched top 15 KEGG terms of MiRNA signature targets.

KEGG Items	Items_Details	No. of genes	FDR
hsa04010	MAPK signaling pathway	13	4.14E-05
hsa04660	T cell receptor signaling pathway	4	7.86E-05
hsa05160	Hepatitis C	4	7.86E-05
hsa05211	Renal cell carcinoma	7	8.57E-05
hsa04370	VEGF signaling pathway	5	8.89E-05
hsa04144	Endocytosis	10	9.18E-05
hsa04062	Chemokine signaling pathway	5	9.22E-05
hsa04910	Insulin signaling pathway	3	9.85E-05
hsa05223	Non-small cell lung cancer	3	9.85E-05
hsa04012	Endometrial cancer	3	9.85E-05
hsa04662	B cell receptor signaling pathway	3	9.85E-05
hsa05215	Prostate cancer	3	9.85E-05
hsa05218	Melanoma	3	9.85E-05
hsa04530	Tight junction	3	9.85E-05
hsa05200	Pathways in cancer	6	0.000105

No.: number; FDR: false discovery rate

## Discussion

CSCC is one of the most common gynecological cancers that affect the health of women [1, 2]. In addition, the 5-year overall survival rate is about 80% [7, 28]. Even so, it is needed to understand the pathological mechanism and find potential survival related genes in the development of CSCC. In this study, we found a miRNA signature including hsa-mir-642a and hsa-mir-378c in CSCC, which could be a valuable tool in guiding treatment decisions for CSCC.

Hsa-mir-642a, a primate-specific miRNA, is a tumor suppressor. It is reported that hsa-mir-642a is differentially expressed in lung cancer cells [29]. Interaction of hsa-mir-642a-5p and Linc00974 can increase the expression of keratin 19 and activate Notch and TGF-β signaling pathways, which will increase the proliferation and invasion of hepatocellular carcinoma [30]. It is found that hsa-mir-642a is over expressed in the pediatric embryonal central nervous system neoplasm that is regarded as a prognostic parameter of patients [31]. In addition, the abnormal expression of hsa-mir-642a in myeloma cell lines significantly decreased protein levels of DEP domain containing MTOR interacting, which caused dedifferentiation of myeloma cells [32]. It is noteworthy that hsa-mir-642a is associated with cervical cancer prognosis [33]. Herein, we also found that hsa-mir-642a was related to survival time of patients with CSCC. Furthermore, cryptochrome circadian clock 2 (*CRY2*) was one of the target genes of hsa-mir-642a. Cryptochrome 2 is circadian clock gene and the hypermethylation of *CRY2* is involved in DNA recombination and repair in long-term shift-workers [34]. It has been demonstrated that the genetic variation of *CRY2* is related to metabolic characteristics of type 2 diabetes [35, 36]. In addition, *CRY2* has been suggested to act as a modulator in the development of cancer [37]. The expression level of *CRY2* in ovarian cancer is remarkably lower than those in normal ovary [38]. The polymorphism in *CRY2* gene has been frequently found associated with increased risk or recurrence of breast and endometrial cancers [39, 40]. Our result showed that hsa-mir-642a was significantly associated with survival time of CSCC and could be a diagnostic and prognostic marker of CSCC.

It is reported that the expression of hsa-mir-378c may enhance cell survival and tumor growth [41]. It has been found that hsa-mir-378c is associated with Stage I and Stage II colon cancer compared with normal controls [42]. Additionally, the expression of hsa-mir-378c is significantly down-regulated in osteosarcoma, intrahepatic cholangiocarcinoma and advanced stage gastric cancer [43–45]. It is worth mentioning that hsa-mir-378c is the member of protective miRNA signatures and correlated with cervical cancer prognosis [33]. In this study, we found that hsa-mir-378c was one of the members of miRNA signatures in the CSCC survival analysis. Moreover, myelin regulatory factor (*MYRF*) was one of the target genes of hsa-mir-378c. *MYRF* is a myelin-associated gene and acts as a key transcription factor for oligodendrocyte differentiation and central nervous system myelination. [46–48]. In addition, it is the target gene of hsa-mir-423-5p and involved in the immune response or injury in the retina [49]. *MYRF* may be involved in the nervous and immune of CSCC. In a word, hsa-mir-378c played a crucial role in the CSCC and could be a diagnostic and prognostic marker in the development of CSCC.

According to the functional annotation analysis of miRNA signature targets, MAPK signaling pathway (FDR = 4.14E-05), VEGF signaling pathway (FDR = 8.89E-05) and endocytosis (FDR = 9.18E-05) were significantly enriched signal pathways that covered most genes. It has been shown that p38 MAPK is involved in a number of cellular processes, including cell survival and death [50, 51]. It is found that human papillomavirus (HPV) 16 E2 can induce apoptosis by inhibiting p38 MAPK/JNK signal pathway in CSCC, which is important for the in vitro growth and migration of cervical squamous carcinoma cells in response to HPV 16 E2 treatment [52]. VEGF has been identified as angiogenesis regulator and may be important to restrict tumor growth, progression and metastasis. Vascular proliferation is a characteristic of cervical cancer and high density of microvessels indicates a worse prognosis of the disease [53]. Tjalma W et al found that the expression level of VEGF was high in cervical cancers [54]. It is suggested that VEGF could stimulate tumor cell proliferation in the early stages and may be responsible for tumorigenesis of cervical cancer [55]. In addition, it has been demonstrated that VEGF could be the predictive biomarker for monitoring the recurrence of cervical cancer [56]. The endocytosis process is involved in regulating various biological process including cell cycle and apoptosis in cancer cells [57, 58]. It has been reported that endocytosis is associated with CSCC-specific alternative splicing events [59]. This suggested that MAPK, VEGF and endocytosis signal pathways may play an important role in CSCC. Inhibition of these signal pathways might be a useful therapeutic strategy for CSCC.

## Conclusions

In summary, we have identified and successfully validated a 2-miRNA signature of hsa-mir-642a and hsa-mir-378c in patients with CSCC. The signature adds to the potential predictive role in the survival time of CSCC patients. Therefore, we can detect the expression of hsa-mir-642a and hsa-mir-378c in the blood to predict the survival time of patients with CSCC, which will improve the clinical outcome for patients with CSCC.
